# Changes in physical fitness and body composition of pilot cadets before and after a process of directed flight preparation

**DOI:** 10.1186/s13102-022-00547-6

**Published:** 2022-08-10

**Authors:** Adam Prokopczyk, Zbigniew Wochyński

**Affiliations:** 1 Department of Sports and Defence Education, Faculty of Sport Sciences, Poznan University of Physical Education, Poznan, Poland; 2 Department of Air Transport Safety, Polish Air Force University, Dęblin, Poland

**Keywords:** Directed training process, Aviation Synthetic Efficiency Test, Body composition, Motor type, Cadet pilots, Physical fitness

## Abstract

**Objectives:**

The aim of the study was to check the changes in the level of physical fitness and body composition after a directed training process in cadets—pilots, in relation to control group.

**Material and methods:**

The study involved 29 cadets studying at the Air Force Military Academy in Dęblin. Group A (study group)—second year pilots (n = 17), male, with an average age of 19.94 ± 1.3 years, studying to become an aircraft pilot, who realized 35-h directed training process based on the Aviation Synthetic Efficiency Test (ASET) and group B (control group)—second year in the field of ground navigation (n = 12), male, with an average age of 19.83 ± 1.27 years, completing the standard physical education process. In both groups, the fitness tests and physiological studies were conducted twice time: before starting the training process—study I; after the training process—study II. Fat mass (FM), fat-free mass (FFM), muscle mass (MM), total body water (TBW), extracellular water (ECW) and intracellular water (ICW) were measurement with using the bioimpedance method with using body composition analyzer the AKERN 101 type BIA 101SE.

**Results:**

In group A in study II, fitness was at a good level, while in group B it was below the standard expected for pilots. There was statistically significant decrease in fat mass (FM) and increase in fat-free mass (FFM), muscle mass (MM), total body water (TBW) in group A compared to group B. In study II, group A showed no significant correlation between ASET and FM, FFM, MM, TBW while group B showed statistically significant correlations.

**Conclusions:**

The results obtained in study II showed an increase in directed physical fitness in groups A and B, as measured by ASET. In both groups A and B, the training process decreased FM and increased FFM, MM, and TBW, but a greater effect of these changes was observed in group A.

## Introduction

The directed training process is one of the most important elements in preparing pilots for flight. This preparation consists in the implementation of a training process by pilots to master the ability to perform the anti-overload maneuver (maneuver-1) using skeletal muscles (lower limbs, upper limbs and abdominal muscles). Electromyographic studies have shown that the following skeletal muscles are involved in the anti-overload maneuver: m. latissimus dorsi, m. intercostalis, m. buccinator m. sternocleidomastoideus, diaphragma, m. pectoralis major [1] and m. rectus femoris, m. rectus abdominis, m. erector spinae [2]. Among the negative factors of flight, the most frequent were situations requiring rapid reaction, especially accelerations (overloads) of +Gz type (head–legs direction) [3, 4]. Therefore, the modern process of directed fitness preparation of pilots requires monitoring of physical loads (to ensure optimal load), appropriate selection and motor adaptation (predisposition) in the pilot's working environment. The Aviation Synthetic Efficiency Test (ASET) and an analytical test consisting of a 40 m run, a 100 m run, a 1000 m run, and a pull-ups were used to achieve this goal [5–8]. Determination of baseline motor predispositions was necessary to identify the motor types that would achieve maximum performance while completing the ASET [7]. Determination of motor predispositions was also used to determine the optimal load for pilots in the training process, ensuring a high level of motor coordination [6, 8, 9] and to prepare for the next stage of special training [10]. The pilot's directed training process loaded the skeletal muscles required to perform maneuver-1, and thus induced physiological changes in the body, such as a change in heart rate and changes in body components after its execution. Moreover, changes in body composition components could extend the diagnostic value of pilots' directed flight preparation [5, 11–13]. They were an important factor in modifying a pilot's diet [14], as well as the intensity (load) of exercise. These factors had a significant impact on the safety and effectiveness of the flight mission [15].

To date, the relationship between body components and directed fitness preparation of pilots for flight has not been analyzed. Therefore, a study was undertaken to analyze the magnitude of changes in body components such as fat mass (FM), fat-free mass (FFM), muscle mass (MM), total water content (TBW), extracellular water content (ECW), and intracellular water content (ICW) before and after a directed training process involving skeletal muscles responsible for performing the anti-overload maneuver.

The authors in this study hypothesized that the directed training process of the pilot would improve fitness levels as measured by the Aviation Synthetic Efficiency Test (ASET) and decrease fat mass and increase fat-free mass and muscle mass in the study group relative to the control group.

The authors also hypothesized that the correlations that existed between body components and the Aviation Synthetic Efficiency Test (ASET) and analytical fitness test would allow assessment of the effects of the training process on motor skills that may influence changes in fat mass (FM), fat-free mass (FMM), and muscle mass (MM) in the study group relative to the control group.

## Material and methods

### Subject

The study included 29 cadets in two male groups studying at the Air Force Military Academy in Dęblin. Group A (study; n = 17) was represented by cadets with an average age of 19.94 ± 1.3 years studying to become an aircraft pilot. Group B (control; n = 12) consisted of cadets with an average age of 19.83 ± 1.27 years studying ground navigation. In both groups fitness and physiological tests were performed twice: before the training process—study I; after the training process—study II. The following somatic parameters were examined: body mass, body height and BMI. In group A in study I: body mass was 76.32 ± 9.37 [kg], body height was 178.63 ± 6.74 [cm] and BMI 23.94 ± 2.83 [kg m^−2^], while study II showed a body mass of 76.7 ± 9.84 [kg] and a BMI of 23.97 ± 2.64 [kg m^−2^]. In group B, the somatic parameters examined in study I, were as follows: body mass 79.65 ± 12.10 [kg], body height 181.66 ± 7.93 [cm] and BMI 24.04 ± 2.45 [kg m^−2^], while in study II: body mass 81.1 ± 11.06 [kg], and BMI 24.5 ± 2.24 [kg m^−2^]. Body height was unchanged in both studies, in both groups. In group A and B between study I and II in body mass and BMI there was no significant difference. Using Student's t-test there was a difference between groups A and B for body mass (*p* = 0.41), and BMI (*p* = 0.92) in study I and in study II respectively (*p* = 0.27) and (*p* = 0.57).

### Ethical issue

The investigators obtained the approval of the Bioethics Committee, at the Medical University of Poznan, issued on May 15, 2019, with the number 610/19.

### Physiological study

Heart rate (HR) and body components were measured twice in both groups in study I and II. Analysis of the dynamics of HR changes was recorded before and after ASET using POLAR—TEAM 2 system. POLAR—TEAM 2 system was also used during the training unit to monitor the training intensity. Cadets during study I and study II was wearing directly on center the chest a strap with wireless transmitter. Each strap with the transmitter was connected to the POLAR TEAM 2 system from which the HR values were read. Based on that measures was given the intensity range of the training process, HR before execiution ASET and HR after execiution ASET.

### Analysis of body components

Measurement was performed with an AKERN type BIA 101SE body composition analyzer according to the guidelines for body components issued by the European Society of Parenteral and Enteral Nutrition (ESPEN). During the analysis of body components, the subject was in a supine position with his limbs at a 30-degree deviation from the body axis. Electrodes were adhered to the skin on the hand and on the foot. On the hand, they were stuck on the medial surface between the wrist joint and the third metacarpophalangeal joint, 4 cm from the metacarpophalangeal joint. On the foot, on the medial top part of the foot, between the ankle and shin joint and the third metatarsophalangeal joint, at a distance of 7 cm from the metatarsophalangeal joint. Before sticking the electrodes, the skin was washed with gauze and disinfectant [16].

### Fitness tests

An analytical fitness test was conducted to assess the level of general fitness. This test consisted of 40 m, 100 m and 1000 m run and pull-ups. It was conducted before and after the training process. Time measurement during the running trials was performed with an accuracy of 0.01 s. The pull-ups on the bar were performed with an overhand grip from an overhanging position, keeping the elbow joints straight. The subject had to pull up to such a height that his chin was above the bar in order to be considered a correctly performed repetition. The Aviation Synthetic Efficiency Test (ASET) was used to assess the cadets' level of directed physical fitness.

### Aviation Synthetic Efficiency Test (ASET)

**Fig. 1:**
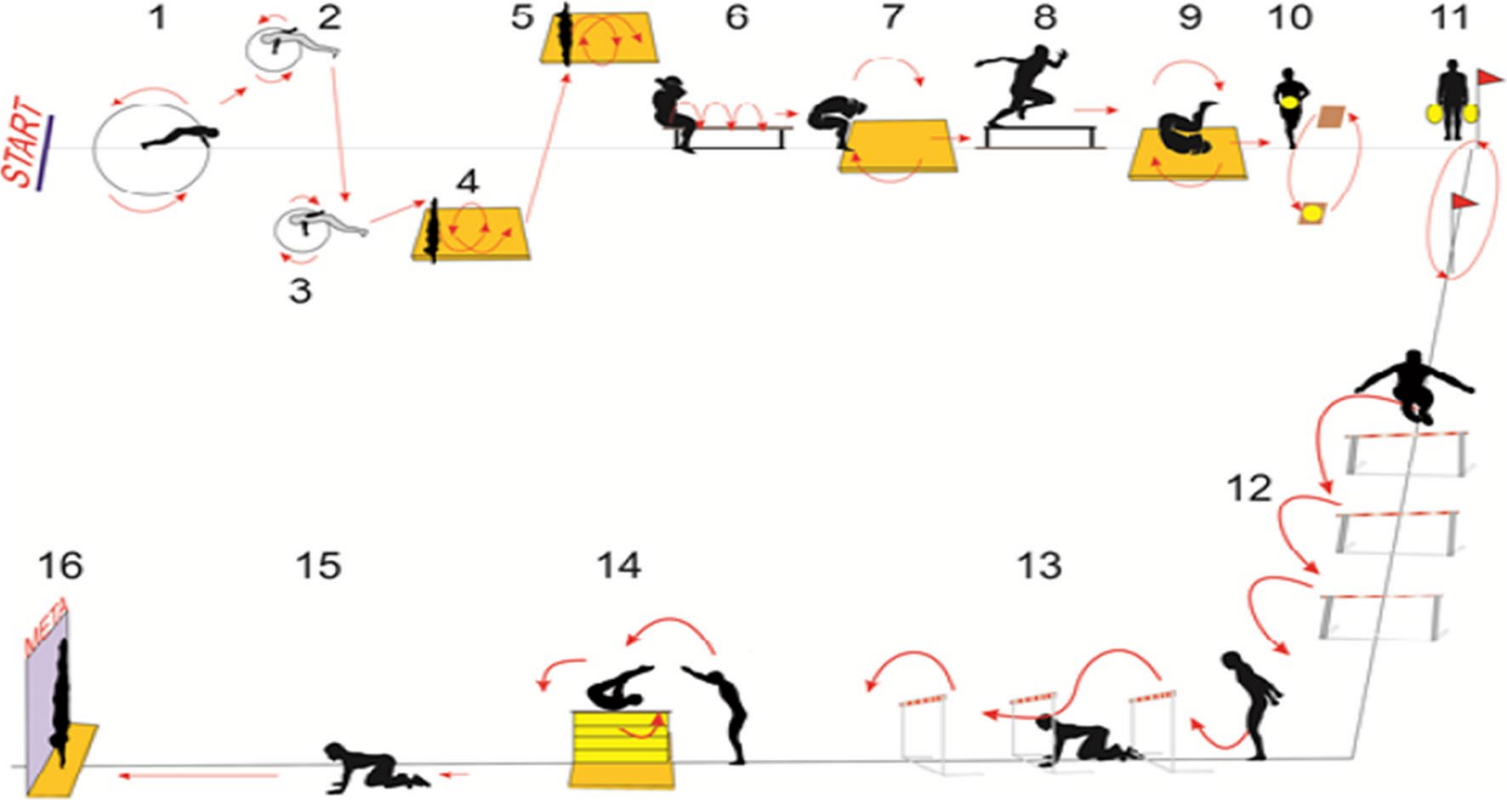
Diagram of Aviation Synthetic Efficiency Test [5, 7]

This is a test consisting of 16 stations and is performed in a timed manner. All stations included in the test are located at a distance of 60 m. The test includes such fitness elements as speed, strength, endurance, flexibility, and jumping ability (e.g. roll over, rolling, jumping, and numerous changes in body position in relation to the ground) (Fig. 1). It is a complex test in terms of difficulty of execution, with high intensity and short work volume. This test diagnoses the subjects in terms of ability to adapt motor activities to changing conditions and situations (orientation), quick reaction, balance and motor adaptation to the pilot's work environment [5, 6]. The exercise stations are set in the right order in such a way that they provide a stimulus of influence for those muscle parts that are involved in the anti-overload maneuver. The following scale is used to evaluate the result (time) obtained during the test.

#### Assessment criteria

Time and assessment standards for men when performing ASET:

- 43 s—very good grade;
- 45 s—good grade;
- 47 s—satisfactory grade.

### Study group training program

The study group was implementing a directed training program as a didactic element of the "pilot" course at the Air Force Military Academy in Dęblin. The training program included 35 training hours and was conducted over a period of 60 days. Training unit included 2 training hours and was 90 min long. In every week were two training sessions. The purpose of the directed training of pilots is to improve the level of physical fitness by mastering physical exercises affecting the muscle groups involved in the execution of the muscle-tensioning maneuver (increasing the tolerance of acceleration +Gz) and improving spatial orientation, agility and motor coordination [8]. Each training session included a 20-min general warm-up. The main part of trainings included speed-strength, strength-endurance, speed-agility exercises. The program included an obstacle courses running, jump exercises, forward and backward flips, balance exercises with the use of devices, exercises with an external load. The final part of trainings included stretching exercises for all parts of the body. In the main part of each trainings cadets had 3 series of exercises. In each series was 8–9 exercises and 3 min of rest between each series. Time of each exercise was 15–20 s of maximum intensity. After every exercise cadets had 25–35 s of rest. During rest cadets did only breathing exercises. Duration of exercise and rest was regulated by the teacher. The stage of directed physical preparation of pilots for flying in high-maneuverability aircraft is preceded by training on Special Aviation Gymnastics Instruments (SAGI) [17]. Based on the heart rate index, the trainings aimed at shaping the directed physical fitness of the pilot were conducted in the zone of aerobic-anaerobic metabolism with the predominance of anaerobic metabolism. The average intensity level of a given training unit measured HR was maintained in the range of 140–170 bpm. The training units were conducted using the repetition, interval, circuit, station and stream methods.

### Training program for the control group

The control group followed a standard physical education program designed for Polish Army cadets. The program included a set of general development exercises, team games and elements of hand-to-hand combat. Training unit included 2 training hours and lasted 90 min. The training program included 35 training hours and was conducted over a period of 60 days. Based on the heart rate index the trainings were conducted in the zone of aerobic metabolism. The average intensity level of a given training unit as measured by HR was kept in the range of 130–140 bpm. The training units were conducted using repetition, station and traditional methods.

All subjects were provided with the same living and eating conditions. The cadets received a standard diet according to the principles of mass nutrition. Daily rations consisted of an average of 4500 kcal, including 150 g of fat (30%), 112.5 g of protein (10%), and 675 g of carbohydrates (60%).The cadets in both groups drank water before and after training, about 1 l in total. They performed the exercises in the ambient temperature of 18 °C.

### Statistical analysis

Descriptive statistics were used in the study by calculating for study I and II arithmetic mean (M), standard deviation (SD); for the study group (group A) and the control group (group B). The results of study I and II were subjected to normality distribution analysis using Shapiro–Wilk Test. The homogeneity of variance was measured with Levene's test. Comparisons of the parameters between groups A versus B and study I and study II was carried out by two-factor analysis of variance Anova with repeated measures. Was used for measured the difference in scores between group A and group B in study I and study II used analysis of variance the Tukey's test (HSD) post hoc pairwise. The effect size between fitness tests and body components at study I and study II was assessed using Hedges’ag test for dependent samples. The effect size between groups was assessed using Hedges’ag test for independent samples. Pearson’s (r)-correlation between body components and physical fitness tests was calculated. The obtained calculation values were considered statistically significant with *p* < 0.05. Statistical analysis was performed using the Statistica 13.3 program.

## Results

The results in Table 1 in group A after the preparation period (study II) showed a statistically significant lower HR after ASET, better performance in the 100 m run, worse performance in the 1000 m run and an increase in the number of pull-ups compared to the values before the training process (study I). In addition, in group A, the study showed statistically significant changes in the composition of body components in the increase of MM [kg] and TBW [kg] values in study II compared to study I. In group B, study II showed statistically significant lower HR after completing ASET, better performance in ASET, better performance in 40 m run, better performance in 100 m run, worse performance in 1000 m run and increase in number of pull-ups compared to study I (Table 1). The other results showed no statistically significant changes.

**Table 1:** Comparison of motor skills, heart rate and body components, between study I and II in groups A (n = 17) and B (n = 12)

Variable	M ± SD		Hedges g test	F	*p*
	Study I	Study II			
ASET [s]					
Group A	45.54 ± 2.59	44.89 ± 4.08	0.19	0.30	0.54
Group B	53.40 ± 2.73	47.40 ± 4.03	1.74	18.18	< 0.0001
HR before execiution ASET [bpm]					
Group A	81.12 ± 13.16	85.53 ± 8.89	0.39	1.31	0.27
Group B	80.17 ± 13.74	90.75 ± 11.41	0.83	4.21	0.05
HR after execiution ASET [bpm]					
Group A	186.59 ± 6.74	180.12 ± 13.96	0.59	2.99	< 0.05
Group B	190.08 ± 4.50	185.67 ± 6.11	0.82	4.06	< 0.05
40 m run [s]					
Group A	5.69 ± 0.33	5.79 ± 0.65	0.19	0.29	0.58
Group B	5.77 ± 0.37	5.43 ± 0.36	0.93	17.65	< 0.0001
100 m run [s]					
Group A	13.74 ± 0.84	13.02 ± 0.70	0.93	7.47	< 0.05
Group B	13.72 ± 0.56	12.83 ± 0.73	1.36	11.29	< 0.002
1000 m run [s]					
Group A	218.65 ± 34.59	252.03 ± 23.98	1.12	10.69	< 0.01
Group B	212.92 ± 12.49	260.94 ± 29.80	2.10	26.51	< 0.0001
Pull-ups on the bar					
Group A	11.29 ± 2.95	13.94 ± 2.28	1.00	8.56	< 0.01
Group B	10.25 ± 3.08	12.92 ± 1.93	1.03	6.46	< 0.01
FM [kg]					
Group A	11.95 ± 3.80	10.58 ± 5.13	0.30	0.98	0.18
Group B	15.44 ± 3.00	14.53 ± 4.63	0.23	1.20	0.22
FM [%]					
Group A	15.56 ± 4.09	13.31 ± 4.97	0.49	2.16	0.09
Group B	19.48 ± 1.69	17.95 ± 4.24	0.47	1.43	0.16
FFM [kg]					
Group A	64.55 ± 7.05	66.22 ± 5.78	0.25	1.66	0.18
Group B	64.09 ± 9.31	65.08 ± 7.81	0.11	0.67	0.48
FFM [%]					
Group A	84.34 ± 4.23	86.77 ± 5.00	0.52	2.34	0.07
Group B	81.08 ± 1.81	82.06 ± 4.25	0.30	0.57	0.43
MM [kg]					
Group A	43.93 ± 4.63	46.24 ± 4.36	0.51	3.86	< 0.02
Group B	44.59 ± 6.80	44.77 ± 5.47	0.03	0.10	0.86
MM [%]					
Group A	58.50 ± 2.74	60.57 ± 4.02	0.60	3.15	0.05
Group B	56.34 ± 1.66	56.45 ± 3.06	0.04	0.01	0.90
TBW [kg]					
Group A	46.39 ± 5.02	48.44 ± 4.23	0.44	2.84	< 0.05
Group B	46.66 ± 6.56	47.59 ± 5.70	0.15	1.13	0.32
TBW [%]					
Group A	61.77 ± 3.04	63.45 ± 3.67	0.49	2.41	0.08
Group B	59.05 ± 1.20	60.02 ± 3.15	0.40	1.41	0.25
ECW [kg]					
Group A	21.86 ± 5.80	19.48 ± 1.97	0.55	2.64	0.08
Group B	23.22 ± 7.44	19.64 ± 2.42	0.64	2.54	0.10
ECW [%]					
Group A	40.85 ± 1.84	40.19 ± 2.29	0.31	093	0.29
Group B	40.59 ± 1.46	41.30 ± 1.73	0.44	2.29	0.08
ICW [kg]					
Group A	31.68 ± 8.75	28.90 ± 2.87	0.42	1.57	0.19
Group B	34.46 ± 12.18	27.90 ± 3.48	0.73	3.22	0.06
ICW [%]					
Group A	58.94 ± 1.75	58.97 ± 3.21	0.01	0.001	0.97
Group B	59.60 ± 1.73	58.61 ± 1.73	0.57	3.95	0.06

*M* ± *SD* mean, standard deviation, *Group A* group of cadets pilots, *Group B* control group, *Study I* study before the training process, *Study II* study after the training proces, *ASET* Aviation Synthetic Efficiency Test, *FM* fat mass, *FFM* free fat mass, *MM* muscle mass, *TBW* total body water, *ECW* extracellular water, *ICW* intracellular water

The results in Table 2 showed a statistically significant difference in study I between group A and B in ASET [s], FAT [kg and %], FFM [%], MM [%], TBW [%] with Hedges’g effect size value in favor of group A. In study II, there was a statistically significant difference in FM [kg and %], FFM [%], MM [%], TBW [%] between group A and B with a value of Hedges’g effect size in favor of group A (Table 2). The other results showed no significant change.

**Table 2:** Comparison of motor skills, heart rate, and body components, between groups A (n = 17) and B (n = 12) in study I and II

Variable	M ± SD		Hedges g test	F	*p*
	Group A	Group B			
ASET [s]					
Study I	45.54 ± 2.59	53.40 ± 2.73	2.97	62.03	< 0.0001
Study II	44.89 ± 4.08	47.40 ± 4.03	0.61	2.68	0.11
HR before execiution ASET [bpm]					
Study I	81.12 ± 13.16	80.17 ± 13.74	0.07	0.03	0.85
Study II	85.53 ± 8.89	90.75 ± 11.41	0.52	1.91	0.17
HR after execiution ASET [bpm]					
Study I	186.59 ± 6.74	190.08 ± 4.50	0.58	2.44	0.12
Study II	180.11 ± 13.96	185.67 ± 6.11	0.48	1.65	0.20
40 m run [s]					
Study I	5.69 ± 0.33	5.77 ± 0.35	0.23	0.40	0.53
Study II	5.79 ± 0.65	5.43 ± 0.36	0.65	2.98	0.09
100 m run [s]					
Study I	13.74 ± 0.84	13.72 ± 0.56	0.03	0.01	0.92
Study II	13.02 ± 0.70	12.83 ± 0.73	0.26	0.49	0.48
1000 m run [s]					
Study I	218.65 ± 34.59	212.92 ± 12.49	0.20	0.29	0.58
Study II	252.03 ± 23.98	260.94 ± 29.80	0.48	0.79	0.38
Pull-ups on the bar					
Study I	11.29 ± 2.95	10.25 ± 3.08	0.34	0.84	0.36
Study II	13.94 ± 2.28	12.92 ± 1.93	0.47	1.60	0.21
FM [kg]					
Study I	11.95 ± 3.80	15.44 ± 3.01	0.99	7.01	< 0.02
Study II	10.58 ± 5.13	14.53 ± 4.63	0.80	4.51	< 0.05
FM [%]					
Study I	15.56 ± 4.09	19.48 ± 1.69	1.17	9.73	< 0.005
Study II	13.31 ± 4.97	17.95 ± 4.24	0.99	6.88	< 0.02
FFM [kg]					
Study I	64.55 ± 7.05	64.09 ± 9.32	0.05	0.02	0.88
Study II	66.22 ± 5.78	65.08 ± 7.81	0.17	0.20	0.65
FFM [%]					
Study I	84.34 ± 4.23	81.08 ± 1.81	0.94	6.26	< 0.02
Study II	86.77 ± 5.00	82.06 ± 4.25	1.00	7.02	< 0.02
MM [kg]					
Study I	43.93 ± 4.63	44.59 ± 6.80	0.11	0.09	0.75
Study II	46.24 ± 4.36	44.77 ± 5.47	0.30	0.64	0.42
MM [%]					
Study I	58.50 ± 2.74	56.34 ± 1.66	0.91	5.91	< 0.05
Study II	60.57 ± 4.02	56.45 ± 3.06	1.12	8.88	< 0.01
TBW [kg]					
Study I	46.39 ± 5.02	46.66 ± 6.56	0.04	0.01	0.90
Study II	48.44 ± 4.23	47.59 ± 5.70	0.17	0.21	0.64
TBW [%]					
Study I	61.77 ± 3.04	59.05 ± 1.20	1.16	8.56	< 0.01
Study II	63.45 ± 3.67	60.02 ± 3.15	0.99	6.88	< 0.02
ECW [kg]					
Study I	21.86 ± 5.80	23.22 ± 7.44	0.20	0.30	0.58
Study II	19.48 ± 1.97	19.64 ± 2.42	0.07	0.04	0.84
ECW [%]					
Study I	40.85 ± 1.84	40.59 ± 1.46	0.15	0.16	0.68
Study II	40.19 ± 2.29	41.30 ± 1.73	0.53	2.02	0.16
ICW [kg]					
Study I	31.68 ± 8.75	34.46 ± 12.18	0.27	0.51	0.47
Study II	28.90 ± 2.87	27.90 ± 3.48	0.32	0.71	0.40
ICW [%]					
Study I	58.94 ± 1.75	59.60 ± 1.73	0.37	0.99	0.32
Study II	58.97 ± 3.21	58.61 ± 1.73	0.13	0.12	0.72

*M* ± *SD* mean, standard deviation, *Group A* group of cadets pilots, *Group B* control group, *Study I* study before the training process, *Study II* study after the training proces, *ASET* Aviation Synthetic Efficiency Test, *FM* fat mass, *FFM* free fat mass, *MM* muscle mass, *TBW* total body water, *ECW* extracellular water, *ICW* intracellular water

In group A, study I revealed significant correlation results between BM and BH, body components and motor skills (40 m, 100 m and 1000 m run and pull-ups) and ASET. A significant correlation was observed between BH and 100 m run (*p* < 0.01). Significant correlation was also observed between 1000 m distance and MM [kg], ECW [%], ICW [%]. In group A of study II, correlations between body composition components and motor skills were not significant (Table 3).

**Table 3:** Pearson's (r)-correlation between body mass and height, body components and motor skills in group A before the training process (study I) and after the training process (study II)

Parameters	BH [cm]	BM [kg]	FM [kg]	FM [%]	FFM [kg]	FFM [%]	MM [kg]	MM [%]	TBW [kg]	TBW [%]	ECW [kg]	ECW [%]	ICW [kg]	ICW [%]
ASET [s]														
Study I	0.07	− 0.08	− 0.10	− 0.10	0.14	0.07	0.01	0.16	− 0.01	0.08	0.21	− 0.12	0.22	0.08
Study II	− 0.02	0.13	0.16	0.12	0.09	− 0.12	0.04	− 0.13	0.09	− 0.09	0.15	0.11	0.02	− 0.08
40 m run [s]														
Study I	0.25	− 0.16	− 0.14	− 0.06	− 0.07	0.04	− 0.16	0.17	− 0.20	0.05	− 0.22	− 0.19	− 0.15	0.16
Study II	− 0.43	− 0.23	− 0.35	− 0.40	− 0.08	0.40	− 0.01	0.45	− 0.08	0.42	− 0.19	− 0.21	0.15	0.24
100 m run [s]														
Study I	0.60#	− 0.10	− 0.12	− 0.11	0.15	0.05	− 0.02	0.26	− 0.08	0.08	0.28	− 0.29	0.36	0.23
Study II	− 0.15	0.07	0.02	− 0.05	0.10	0.05	0.10	0.06	0.11	0.07	0.08	− 0.01	0.10	0.08
1000 m run [s]														
Study I	0.11	− 0.33	− 0.19	− 0.05	− 0.20	0.03	− 0.47*	− 0.20	− 0.35	0.04	− 0.18	0.53**	− 0.30	− 0.59***
Study II	− 0.27	− 0.17	− 0.19	− 0.23	− 0.13	0.23	− 0.12	0.17	− 0.12	0.24	− 0.09	0.03	− 0.12	− 0.20
Pull-ups on the bar														
Study I	− 0.05	− 0.34	− 0.37	− 0.33	− 0.30	0.33	− 0.31	0.18	− 0.23	0.33	− 0.026	0.33	− 0.33	− 0.32
Study II	− 0.07	− 0.10	− 0.16	− 0.09	− 0.03	0.20	0.11	0.26	− 0.03	0.08	− 0.30	− 0.46	0.16	0.22

*ASET* Aviation Synthetic Efficiency Test, *BH* body height, *BM* body mass, *FM* fat mass, *FFM* free fat mass, *MM* muscle mass, *TBW* total body water, *ECW* extracellular water, *ICW* intracellular water

^*^Borderline statistical significance (*p* = 0.05)

^**^Statistically significant correlation (*p* < 0.05)

^***^Statistically significant correlation (*p* < 0.02)

#Statistically significant correlation (*p* < 0.01)

In group B before the training process, there was a statistically significant correlation between 40 m run and ECW [kg] and ICW [kg] content. A significant correlation was also observed between pull-ups with ECW [%] and ICW [%] content. After the training process in group B, a statistically significant correlation was observed between ASET and BM [kg], FM [kg], FFM [kg], MM [kg], TBW [kg], ECW [kg], ICW [kg]. Statistically significant correlation was found between 100 m run and BH [cm], BM [kg], FFM [kg], TBW [kg], ECW [kg] as well as between 1000 m run and FFM [kg], MM [kg], ECW [kg]. Statistically significant correlation was also found between pull-ups and BM [kg] and MM [kg] (Table 4).

**Table 4:** Pearson's (r)-correlation between body mass and height, body components and motor skills in group B before the training process (study I) and after the training process (study II)

Parameters	BH [cm]	BM [kg]	FM [kg]	FM [%]	FFM [kg]	FFM [%]	MM [kg]	MM [%]	TBW [kg]	TBW [%]	ECW [kg]	ECW [%]	ICW [kg]	ICW [%]
ASET [s]														
Study I	0.29	0.17	0.01	− 0.20	0.17	0.23	0.16	0.19	0.17	0.25	0.15	0.05	0.13	0.03
Study II	0.52	0.78##	0.71#	0.51	0.69***	− 0.51	0.69***	− 0.45	0.69***	− 0.51	0.65**	− 0.10	0.69***	0.11
40 m run [s]														
Study I	0.06	− 0.13	− 0.21	− 0.23	− 0.11	− 0.05	− 0.15	− 0.25	− 0.08	0.16	− 0.57*	0.33	− 0.59**	− 0.46
Study II	0.20	0.01	− 0.01	− 0.05	0.05	0.05	0.02	− 0.01	0.05	0.05	0.10	0.14	0.01	− 0.13
100 m run [s]														
Study I	0.45	0.11	− 0.04	− 0.29	0.15	0.17	0.10	− 0.12	0.17	0.30	− 0.15	0.46	− 0.21	− 0.43
Study II	0.70**	0.61**	0.57	0.36	0.62**	− 0.37	0.54	− 0.50	0.62**	− 0.36	0.73#	0.40	0.51	− 0.39
1000 m run [s]														
Study I	− 0.24	− 0.10	− 0.14	− 0.22	− 0.03	0.17	− 0.03	0.12	− 0.02	0.24	− 0.08	0.08	− 0.09	− 0.05
Study II	0.22	0.48	0.22	− 0.04	0.61**	0.04	0.59**	0.02	0.61**	0.04	0.61**	0.07	0.57	− 0.08
Pull-ups on the bar														
Study I	− 0.07	− 0.29	− 0.31	− 0.28	− 0.22	0.06	− 0.29	0.23	− 0.21	0.23	− 0.46	0.66***	− 0.53	− 0.70***
Study II	− 0.20	− 0.66***	− 0.59**	− 0.41	− 0.52	0.41	− 0.53	0.33	− 0.52	0.41	− 0.44	0.20	− 0.53	− 0.19

*ASET* Aviation Synthetic Efficiency Test, *BH* body height, *BM* body mass, *FM* fat mass, *FFM* free fat mass, *MM* muscle mass, *TBW* total body water, *ECW* extracellular water, *ICW* intracellular water

^*^Borderline statistical significance (*p* = 0.05)

^**^Statistically significant correlation (*p* < 0.05)

^***^Statistically significant correlation (*p* < 0.02)

#statistically significant correlation (*p* < 0.01)

##Statistically significant correlation (*p* < 0.005)

In group A in study I there was statistically significant correlation between ASET and pull-ups and in study II between ASET and 40 m run, 100 m run and pull-ups. In group B, a statistically significant correlation was found between ASET and 100 m run in study I and II (Table 5).

**Table 5:** Pearson's (r)-correlation between ASET and motor skills

Parameters	ASET [s]			
	Group A		Group B	
	Study I r	Study II r	Study I r	Study II r
40 m run [s]	0.28	0.49**	0.06	0.11
100 m run [s]	0.33	0.70##	0.63**	0.72#
1000 m run [s]	− 0.24	0.40	0.17	0.26
Pull-ups on the bar	− 0.64#	− 0.61#	0.06	− 0.56

*r* correlation coefficient

^*^Borderline statistical significance (*p* = 0.05)

^**^Statistically significant correlation (*p* < 0.05)

^***^Statistically significant correlation (*p* < 0.02)

#Statistically significant correlation (*p* < 0.01)

##Statistically significant correlation (*p* < 0.005)

## Discussion

Based on the results of the study, it was concluded that the applied training process in group A (study) has an effect on the increase of directed fitness in pilots. This is evidenced by the better result in study II compared to study I. In addition, better fitness may have been influenced by motor predisposition to the pilot's work environment (achieving a score within the accepted evaluation criteria for pilots in Study I). Group B achieved a significant improvement in directed physical fitness in Study II, but below the lower limit of the accepted score. Therefore, a high statistically significant difference was found in Study I between Group A and B in overcoming ASET. Study II showed a significant difference between groups A and B, but not statistically significant. This could indicate that the training program had an effect on directed fitness (as measured by ASET) in group B, but below the accepted evaluation criteria for pilots. In Study II, the difference in directed fitness between the groups was due to the different application of the training programs, and may also have been influenced by motor predisposition in the subjects with respect to body mass. From the analysis of the results of the motor skills, based on the results of the 40 m run, 100 m run, 1000 m run and pull-ups, it can be seen that group A showed speed/strength motor dominance during the training process and group B showed speed dominance. This could be confirmed by significant correlations between ASET scores and 40 m run, 100 m run and pull-ups in group A and correlations between ASET scores and 100 m run in group B. In an earlier study on cadet pilots, it was found that the endurance/speed motor type in the group with body mass up to 73.8 kg, the endurance/strength motor type in the group with body mass above 73.8 kg, and the endurance/strength motor type in the group of all subjects obtained the best results in overcoming the ASET [7]. In the overload centrifuge test conducted on cadet pilots to assess their level of performance of the anti-overload maneuver, it was found that the subjects with endurance/strength abilities achieved the best results. This study was monitored by lipid index, which confirmed this fact [18].In a recent study, it was shown that the correlation between ASET and motor ability scores of subjects is helpful in guiding the optimization of the training process for pilots [6]. Optimization of the training process determines the maximum speed of overcoming ASET [6]. In the study of Wochynski et al. [8], it was shown that obtaining maximum results in the analytical test, i.e. in the 1000 m run, 100 m run and pull-ups resulted in a simultaneous deterioration of the results in overcoming the LSTS due to the reduction (disruption) of motor coordination. Starosta's observed that exceessive the physical load in shaping motor skills may reduce the level of motor coordination [19]. Coordination abilities are more strongly genetically conditioned, and the development of strength and endurance abilities has an exceptionally colliding character. At the same time, their rapid development may inhibit or reduce the development of almost all coordination skills. The fact of exceeding the load in the formation of general physical fitness (e.g. in endurance exercises) may be closely related to the decrease in the level of motor skills and motor abilities necessary for the pilot. The relationship of load magnitude with a decrease in motor coordination has also been observed in other scientific works [20–22]. In group A, the decrease of the score at the distance of 1000 m could be justified by the emphasis on strength and speed training in order to achieve maximum speed in overcoming ASET at lower somatic parameters (height and body mass) than in group B. In group B, the decrease in the 1000 m distance score could be justified by the fact that the training process was less optimized compared to group A. Moreover, the training emphasis was on speed and in accordance with the group's predispositions for this motor ability with higher somatic parameters than in group A. It seems that in both groups the level of running endurance achieved in study I with consideration of somatic characteristics would be an obstacle (interference) in reaching the maximum speed of overcoming ASET presented in study II. In the process of pilot training, running endurance was always the most important element that influenced the level of performance of the anti-overload maneuver. It was shown in pilots that a high level of running endurance reduced the ability to tolerate + Gz accelerations (to perform the anti-overload maneuver) [23, 24]. Running endurance in pilots has been the subject of much discussion and research, but this problem has not been fully resolved. According to the authors of this paper, in addition to the genetic predisposition to motor skills in the subjects, the basis in the pilot's targeted training is the optimization of the impact (load emphasis) on all basic motor skills using ASET. So far, it has been shown that somatic characteristics also had a significant influence on performance in ASET [7]. In a study on cadet pilots, it was found that the group which had the lowest body height (177.03 cm) obtained the best speed on this test [8]. Ziółkowska's study [25] proved that long-leggedness, trunk shape and body height are selection indicators for the pilot profession. The difference in the directed fitness of the studied groups may be due to body build, motor predispositions and the program of the training process. It has also been shown that in different types of motor skills, the body mass of the subjects plays a big role [7]. In the present study, group A had a lower body mass than group B. Noteworthy is the increase in body mass in group B in study II compared to study I. This fact can be associated with the low intensity of endurance exercise and the subjects' predisposition to speed running, which is confirmed by the correlation results in Table 5. However, it was also partly related to the increase in TBW, FFM and MM. In the study conducted, by analyzing the components of body mass, a statistically significant difference was found in study I and II between groups A and B in FM [kg, %], FFM [%], MM [%], BTW [%]. This may indicate the higher intensity of the training process in group A compared to group B. It was observed that as a result of the training process, both groups showed a decrease in FM, increase in FFM, MM, BTW with a greater effect of changes in group A than in group B. A similar trend of changes in body components in relation to body mass and physical fitness in cadet pilots was reported in the study of Kłossowski [26]. It is also worth noting the difference in intensity and the same duration of the training unit between the studied groups, which is likely to confirm that the body of trainees in group A adapts differently than in group B. Probably the training process in group A took place with very little fat burning as no significant correlation between ASET and FM was found. In group B, statistically significant correlations were found between ASET and FM, FFM, TBW, ECW, ICW in study II, which could indicate a moderate intensity of the training process and ongoing exercise adaptation with a high proportion of fat burning. In a previous study [17], a significant relationship was found at the end of the training period in the study group exercising on Special Aviation Gymnastics Instruments (SAGI) between ASET and body components such as: FM, FFM, TBW, ECW, ICW, MM, while the control group showed no significant correlation. It was observed that the difference in intensity with the same duration of training unit affects the process of exercise adaptation of both groups. The body of exercisers in the study group adapted differently than in the control group. In the study group special training program on SAGI, with an average intensity of HR = 109 bpm, a significant correlation was found between ASET and FM in contrast to the control group which followed a higher intensity training program HR = 141 bpm. The occurrence of a significant correlation between ASET and FM could be indicative of the adaptation going on in the study group with the fat burning process [17].

In conclusion, in our study, the performed analysis of body components together with physical fitness show great usefulness to evaluate the level of directed fitness measured by ASET (load of skeletal muscle groups involved in anti-overload maneuver). The correlation between ASET and individual motor abilities (analytical fitness test) is very helpful in diagnosing the impact of the training process on motor abilities and optimizing the training process. The demonstrated relationships between physical fitness and body components in cadet pilots in study I and II including the control group, could provide information about the course of adaptation in directed training. In addition, the present study is important for future research regarding dietary habits, hydration changes, and changes in body composition components in order to achieve optimal motor skills necessary in the extreme conditions of a pilot's work environment.

## Conclusions

The training process in group B (control) in study II had a significant effect on increasing directed fitness (as measured by ASET), which was below the lower limit of the standard accepted for pilots, but it was not greater than group A (study), which was at a good level. In both groups A and B, the training process decreased the percentage of FM and increased the percentage of FFM, MM and TBW, but with a greater effect in favor of group A. This was evidenced by the statistically significant differences between the groups. The magnitude of these changes was influenced in study II by strength and speed motor skills in group A and speed motor skills in group B as evidenced by the correlations found between ASET and motor skills.

The correlations found in study II between body components and ASET and motor skills in group A and B indicated a different course of adaptation in these groups. In group A in study II, the lack of significant correlation between ASET and FM could indicate adaptation with a very low participation of the fat burning process. The significant correlation between ASET and FM in group B could indicate that the adaptation process took place with a high degree of fat burning.

## Data Availability

The datasets generated and/or analysed during the current study are not publicly available due data confidentiality of the training process at the Polish Air Force University but are available from the corresponding author on reasonable request.

## References

[1] Chen HH, Wu YC, Kuo MD (2004). An electromyographic assessment of anti-G straining manoeuvre. Aviat Space Environ Med.

[2] Oksa J, Hamalainen O, Rissanen S, Myllyniemi J, Kuromen P (1996). Muscle strain during aerial combat manoeuvering exercise. Aviat Space Environ Med.

[3] Huttumen K, Keränen H, Väyrynen E, Pääkkönen R, Leino T (2011). Effect of cognitive load on speech prosody in aviation: evidence from military symulator flights. Appl Ergon.

[4] Mohler SR (1972). G effects on the pilot during aerobatics.

[5] Wochyński Z, Krawczyk P, Cur K, Kobos Z (2021). An assessment of physical efficiency in cadet pilots before and after the implementation of a program preparing for flights. Int J Occup Med Environ Health.

[6] Wochyński Z (2021). Evaluation of judo practitioners’ motor performance in relation to the criterion of targeted fitness of pilot cadets after a 6-month training process. Arch Budo.

[7] Wochyński Z, Skrzyńsa-Rękawek J, Pilaczyński P, Kobos Z (2020). The impact of motor predispositions in cadets upon the results of the execution of Aviation-Synthetic Efficency Test. Arch Budo Sci Martial Arts Extreme Sports.

[8] Wochyński Z, Stelęgowski A, Kłossowski M (2010). Application of the Aviation Synthetic Efficiency Test for the purpose of selecting candidates for Air Force Officers College for multi tasks aircraft, type F-16. Pol Prz Med Lot.

[9] Herrador-Colmenero M, Fernández-Vicente G, Ruiz JR (2014). Assessment of physical fitness in military and security forces—a systematic review. Eur J Hum Mov.

[10] Astani AI, Macarie A (2013). The ieal ability profile of the student future military aircraft pilot. Rev Air Force Acad.

[11] Gaździńska A, Baran P, Skibniewski F, Truszczyński O, Gaździński S, Wyleżoł M (2015). The prevalence of overweight and obesity versus the level of physical activity of aviation military academy students. Med Pr.

[12] Bustamante-Sánchez A, Clemente-Suárez VJ (2020). Body composition differences in military pilots and aircrew. Aerosp Med Hum Perform.

[13] Cárdenas D, Madinabeitia I, Vera J (2020). Better brain connectivity is associated with higher total fat mass and lower visceral adipose tissue in military pilots. Sci Rep.

[14] Anyżewska A, Łakomy R, Lepionka T, Szarska E, Maculewicz E, Tomczak A, Bertrandt J (2020). Association between diet, physical activity and Body Mass Index, Fat Mass Index and bone mineral density of soldiers of the Polish Air Cavalry Units. Nutrients.

[15] Rintala H, Häkkinen A, Siitonen S, Kyröläinen H (2015). Relationships between physical fitness, demands of flight duty, and musculoskeletal symptoms among military pilots. Mil Med.

[16] Kyle UG, Bosaeus I, De Lorenzo AD, Deurenberg P, Elia M, Gómez JM, Heitmann BL, Kent-Smith L, Melchior JC, Pirlich M, Scharfetter H, Schols AM, Pichard C (2004). Bioelectrical impedance analysis—part I: review of principles and methods. Clin Nutr.

[17] Wochyński Z, Jędrys R, Stelęgowski A (2010). Methodology of exercises on Special Aviation Gymnastics Instruments.

[18] Wochyński Z, Kowalczuk K, Kłossowski M (2016). Effect of the centrifuge test on Blood Serum Lipid Index of cadet pilots. Ann Agric Environ Med.

[19] Starosta W, Starosta W (2003). Współzależność zdolności kondycyjnych i koordynacyjnych. Motoryczne zdolności koordynacyjne.

[20] Raczek J (2010). Antropomotoryka. Teoria motoryczności człowieka w zarysie.

[21] Sozański H, Sozański H, Czerwiński J, Sadowski J (2013). Nauka o sporcie. Teoria sportu. Teoria treningu. Technologia treningu. Podstawy teorii i technologii treningu sportowego.

[22] Starosta W, Osiński W (1993). Koordynacja ruchowa człowieka. Motoryczność człowieka—jej struktura, zmienność i uwarunkowania.

[23] Kłossowski M, Klukowski K, Markiewicz L. Odrębności treningu wytrzymałościowego pilotów samolotów odrzutowych Medycyna Lotnicza. 1993;1–2(27):71–81.

[24] Whinnery JR, Parnell MJ (1987). The effects of long-term aerobic conditioning on +Gz tolerance. Aviat Space Environ Med.

[25] Ziółkowska E (1995). Body build of military pilots. Phys Educ Sport.

[26] Kłossowski M, Stelęgowski A (2004). Ocena związku między masą i składem ciała a sprawnością fizyczną podchorążych Wyższej Szkoły Oficerskiej Sił Powietrznych. Pol Przegl Med Lotn.

